# Lymphopenia and high Ki-67 expression in peripheral blood CD4+ and CD8+ T cells associate with progressive sarcoidosis

**DOI:** 10.1136/bmjresp-2022-001551

**Published:** 2023-12-13

**Authors:** Susanna Kullberg, Johan Grunewald, Anders Eklund

**Affiliations:** 1 Respiratory Medicine Division, Department of Medicine, Karolinska Institutet, Stockholm, Sweden; 2 Department of Respiratory Medicine, Theme Inflammation and Ageing, Karolinska University Hospital, Stockholm, Sweden

**Keywords:** Sarcoidosis, Systemic disease and lungs, Lymphocyte Biology, Bronchoscopy

## Abstract

**Background:**

Early identification of patients at risk for progressive sarcoidosis may improve intervention. High bronchoalveolar lavage fluid (BALF) lymphocytes and peripheral blood (PB) lymphopenia are associated with worse prognosis. The mechanisms behind are not disentangled, and to date, it is not possible to predict disease course with certainty.

**Objectives:**

Insight into the frequency of T regulatory cells (T_regs_), proliferating CD4+ and CD8+ T cells in BALF and PB in clinically well-characterised patients, may provide clues to mechanisms behind differences in disease course.

**Methods:**

Nineteen treatment-naïve patients with newly diagnosed sarcoidosis were assessed with BAL and PB samples at diagnosis. From the majority, repeated PB samples were collected over a year after diagnosis. The patients were followed for a median of 3 years and clinical parameters were used to classify patients into resolving, chronic progressive and chronic stable disease. Lymphocyte counts, frequency of T_regs_ defined as forkhead box protein 3+ (FoxP3+) CD4+T cells, and proliferating CD4+ and CD8+ T cells assessed with Ki-67 were analysed.

**Results:**

Eleven patients disclosed a chronic stable, and eight a progressive disease course, no one resolved during the study period. In PB, lower number of lymphocytes associated with chronic progressive disease, an increased frequency of Ki-67+CD4+ and CD8+ T cells, and a tendency towards higher percentage of FoxP3+CD4+ T cells compared with chronic stable patients.

**Conclusion:**

A reduction of PB lymphocytes despite increased proliferation of CD4+and CD8+ T cells was observed in patients with chronic active compared with chronic stable sarcoidosis, indicating an increased PB lymphocyte turn-over in patients with deteriorating disease. Measurement of PB T_regs_, Ki-67+CD4+ and Ki-67+CD8+ T cells may help in predicting sarcoidosis disease course.

**WHAT IS ALREADY KNOWN ON THIS TOPIC**

- Peripheral blood (PB) lymphopenia in patients with sarcoidosis associated with worse prognosis. The mechanisms behind are not disentangled, and biomarkers to predict sarcoidosis disease course with certainty are lacking.

**WHAT THIS STUDY ADDS**

- A reduction of PB lymphocytes despite increased proliferation of CD4+ and CD8+ T cells was observed in patients with chronic active compared with chronic stable sarcoidosis, indicating an increased PB lymphocyte turn-over in patients with deteriorating disease.

**HOW THIS STUDY MIGHT AFFECT RESEARCH, PRACTICE OR POLICY**

- Measurement of percentage PB proliferating CD4+ and CD8+ T cells may help in predicting sarcoidosis disease course.

## Introduction

A lymphocytosis due to accumulation of CD4^+^ T cells, and increased production of pro inflammatory cytokines, for example, TNF-α and IFN-γ, are typically seen in the lungs of individuals with sarcoidosis, a systemic granulomatous inflammatory disease. Virtually any organ can be involved but the lungs are most commonly affected. The disease course is highly variable. Patients with the clinical phenotype Löfgren’s syndrome (LS), characterised by an acute onset often come to spontaneous resolution. In contrast, patients with non-LS (non-LS), usually with a more insidious onset, disclose a more heterogeneous disease course with some patients experiencing a progressive disease despite immunosuppressant therapy, others a chronic stable course and some resolve spontaneously.^1^

CD4+T cells play a major role in the pathogenesis of sarcoidosis^1^ and are also implicated in disease outcome. For instance, high bronchoalveolar lavage fluid (BALF) lymphocytes associates with a chronic disease,^2^ and higher percentage replicating BALF Ki-67+CD4+ T cells is reported in patients with active, compared with non-active sarcoidosis and healthy controls, using the proliferation marker Ki-67.^3^ In patients with LS, BALF lymphocyte numbers and percentage normalised after spontaneous resolution.^4^ Increasing evidence points to a contribution also of CD8+T cells in the pathogenesis.^1 5^ In both BALF and peripheral blood (PB), there is a high frequency of CD8+T cells producing IFN-γ compared with healthy controls.^6^ PB CD8+ cytolytic lymphocytes with higher activity are found in increased proportions in non-LS patients (ie, patients at higher risk for chronic disease) than in patients with LS and healthy controls.^7 8^

The exaggerated TH1 inflammation has, at least partly, been explained by a dysregulation of T_regs’_^1 9^ which are identified through expression of FoxP3, and an appropriate function is required to terminate immune responses after antigen eradication. Despite contradictory results about T_reg_ proportions in BALF and PB from sarcoidosis patients, an impaired T_reg_ immunosuppressive function is consistently reported. It is generally held likely that a decreased inhibition of TH1 responses due to dysfunctional T_regs_ contributes to the exaggerated immune reaction.^1 9–12^

As opposed to the lung lymphocytosis, PB lymphopenia is observed in a subset of sarcoidosis patients^13 14^ and is associated with a less favourable prognosis and high inflammatory activity, but the mechanisms behind are not fully understood.^14–16^ Migration of PB lymphocytes to the lung and extrapulmonary organs, as well as a lymphocyte depletion due to increased peripheral destruction have been suggested as explanations for the PB lymphopenia.^16 17^

We hypothesised that studying lymphocytes with focus on T_regs_, proliferating CD4+ and CD8+ T cells in two compartments (blood and lung), at sarcoidosis diagnosis and in follow-up studies, may provide mechanistic clues to clinical phenotype differences, and offer valuable information that could make decisions on treatment better founded.

## Methods

### Characterisation of study subjects and study design

Participants were identified among consecutive patients referred to the Department of Respiratory Medicine, Karolinska University Hospital, Stockholm, Sweden. Nineteen treatment-naive non-smoking patients without signs of other pulmonary, cardiac, chronic inflammatory or metabolic diseases, with newly diagnosed sarcoidosis fulfilling the criteria for sarcoidosis according to the World Association of Sarcoidosis and Other Granulomatous Disorders were included.^18 19^ Nine of the included patients participated in a 3-month training programme as part of another study^20^ (see table 1).

**Table 1:** Baseline characteristics of patients

Parameter	Chronic progressive	Chronic stable	P value
Sex (F/M)	2/6	3/8	0.96
Age	50 (36–51)	44 (42–48)	0.68
Scadding (0/1/II/III/IV)	0/2/4/2/0	0/3/5/2/1	0.97
EPM (no of organs involved)	2.0 (1.75–2.25)	2.0 (1.5–2.5)	0.79
Training (yes/no)	5/3	5/6	0.51
Treatment (yes/no)	4/4	0/11	0.012

Data are presented as n or median (25th–75th percentile). Training denotes patients participating in a 3-month training programme, described in reference number.^21^ Treatment denotes treatment at third follow-up.

EPM, extrapulmonary manifestation; F, female; M, male; Scadding, radiographic extent of sarcoidosis assessed by chest X-ray using Scadding staging system (0–IV).

A general somatic evaluation, including a CT scan of the thorax, chest X-ray, a 12-lead ECG, blood samples for blood cell counts, C reactive protein, ACE, liver and kidney function, electrolytes, calcium, and albumin, was completed. All patients except for one were also screened for hypercalciuria. Bronchoscopy with BAL was performed as previously described^21 22^ and baseline blood samples were drawn within a couple of weeks after referral. The patients were then followed and blood samples were drawn at median 6 months (first follow-up) and 11 months (second follow-up) after baseline sampling. PB lymphocytes were matched as close as possible to the date for BAL. In median, 36 days (range 5–61 days) had passed between sampling for PB lymphocytes and BAL. As reference values at Karolinska University laboratory for absolute numbers of PB lymphocytes changed during the study, and some blood samples for PB lymphocytes were analysed at external laboratories with slightly different reference values (1.0–4.0; 0.8–4.1; 1.0–4.1; 1.1–3.5×10^9^/L), percentage of lower normal limit was calculated. A final clinical evaluation was carried out at median 3 years after baseline (third follow-up). EPM was evaluated as the number of organs involved, defined as a biopsy showing non-necrotising granulomas from the affected organ, obvious symptoms, signs on CT scan or assessment of a specialist in that area. Enlarged lymph nodes were regarded as one organ. Non-resolving disease was defined as remaining signs of disease at third follow-up, evaluated by chest X-ray, lung function, patient symptoms and laboratory signs of inflammation. Patients were further classified as having a chronic progressive disease in cases with worsening pulmonary manifestations, signs of increasing inflammatory activity in laboratory parameters and/or systemic treatment required. Chronic stable disease was defined as remaining pulmonary manifestations without deterioration, no signs of inflammatory activity in laboratory parameters and no systemic treatment required.

### Patient and public involvement

Patients were not involved in the design of the study, setting the research questions or outcome measures. Eligible patients were involved in recruitment. They were informed about the study as well as alternative follow-ups by their treating physician (SK and AE) and took an active part in the decision whether they should be included or not. Patients that were interested in BALF and PB data were informed about those results. The results will be disseminated to wider patient communities at a so-called ‘information day about sarcoidosis for patients’, an event arranged by our unit. For the moment, we have no plans for involving patients directly in disseminating the results to medical caregivers and scientific communities.

### BALF and PBL

Centrifugation was used to separate BALF and PB mononuclear cells. BALF cells were then fixed on cytospin slides and stained with Giemsa for calculation of leucocyte differential count. The percentage of lymphocyte subtypes was measured by triple-laser, eight-colour flow cytometry using a FACS Fortessa X-20 (Becton-Dickinson). The following antibodies were used: CD3 (BD, Pharmingen, California, USA); CD4, CD8, Ki-67 (BD Biosciences, California, USA); FoxP3 (Invitrogen, AH Diagnostics). For subtyping the following gating strategy was used; proliferating CD4+T cells: Ki-67+CD3+CD4+, proliferating CD8+T cells: Ki-67+CD3+CD8+, T_regs_: CD3+CD4+FoxP3+. Total PB lymphocytes were analysed at Karolinska University Hospital laboratory as part of routine clinical follow-up.

### Data analysis

Descriptive statistics were used for calculation of median values, the Mann-Whitney U test for comparisons between groups and Spearman′s rank for correlations. P value significance was set at <0.05. All analyses were performed using Jamovi V.1.1.9.0 software (https://www.jamovi.org).

## Results

### Study subjects

Nineteen patients were included and all of them underwent BAL and baseline PB samples (data for Ki-67 was only available from 17 patients), 8 were classified as having a progressive disease and 11 chronic stable. PB was available from 15 (6 with progressive and 9 with chronic stable disease) and 14 patients (5 with progressive and 9 with chronic stable disease) at first and second follow-up, respectively. All patients participated in the final clinical evaluation, no one had then resolved. Numbers of EPM did not differ between patients with chronic progressive and chronic stable disease. At baseline, first and second follow-ups, all patients were still treatment-naive. Four out of eight patients in the chronic progressive group were treated with immunosuppressant at the final clinical evaluation. Three of the patients not receiving treatment were suggested treatment but refused, and one patient was regarded as not appropriate for treatment due to development of a serious disease not related to sarcoidosis.

For more detailed clinical characteristics, see table 1.

### PB cells

As shown in table 2, none of the examined parameters disclosed any significant change from baseline to first and second follow-ups, respectively. No difference in any of the parameters was observed between patients participating in the 3 months training programme and patients that did not (not shown). Patients with chronic progressive sarcoidosis had lower median lymphocytes (both concentration and percentage of lower limit for normal values) than chronic stable patients at all time points, though not significant at baseline. The median percentage of Ki-67+CD4+ T cells in chronic progressive patients was significantly higher than in chronic stable patients at baseline and second follow-up, and reached borderline significance at first follow-up. Similarly, the median percentage of Ki-67+CD8+ T cells was increased in chronic progressive patients compared with stable patients at all time points, but reached significance only at second follow-up. There was a trend towards increased frequency of FoxP3+CD4+ T cells in chronic progressive compared with chronic stable patients, with borderline significance at baseline and second follow-up.

**Table 2:** Median values (25th–75th percentile) of parameters examined in peripheral blood (PB) and bronchoalveolar lavage fluid (BALF) in patients with chronic progressive (P) and chronic stable (S) sarcoidosis

Parameter	Time point	P (chronic progressive)	S	P value
BALF lympho (/10^6^ L)	Baseline	48.3 (24.5–81)	28.3 (17.9–46.8)	0.21
BALF lympho (%)	Baseline	33.5 (24.2–41.6)	16.5 (13.8–33.3)	0.069
PB lympho (×10^9^/L)	Baseline	1.0 (0.9–1.2)	1.4 (1.0–1.6)	0.14
	1st follow-up*	1.1 (1.0–1.2)	1.5 (1.2–1.6)	0.042
	2nd follow-up*	1.1 (0.8–1.2)	1.4 (1.3–1.7)	0.013
PB lympho (% of ll)	Baseline	112 (85–133)	140 (95.5–160)	0.36
	1st follow-up*	106 (91–120)	150 (120–150)	0.042
	2nd follow-up*	110 (73–120)	130 (127–163)	0.013
% Ki-67+CD4+ T cells	BALB baseine	5.1 (4.6–6.6)	4.3 (3.2–5.4)	0.18
	PB baseline**	4.1 (3.8–4.6)	2.6 (2.5–3.0)	0.002
	PB 1st follow-up	5.0 (3.8–5.7)	3.2 (2.7–4.5)	0.087
	PB 2nd follow-up*	5.1 (4.1–6.0)	2.2 (1.9–3.3)	0.033
% Ki-67+CD8+ T cells	BALF baseline	4.0 (3.3–4.5)	4.1 (3.4–4.8)	0.92
	PB baseline	2.9 (2.4–3.3)	2.0 (1.4–3.2)	0.34
	PB 1st follow-up	2.5 (1.9–3.0)	2.0 (1.8–2.1)	0.41
	PB 2nd follow-up**	3.2 (3.0–3.4)	1.4 (1.3–1.6)	0.004
% FoxP3+CD4 T cells	BALF baseline	5.4 (4.1–6.1)	4.6 (3.3–5.0)	0.30
	PB baseline	6.4 (4.6–7.7)	2.4 (2.1–5.3)	0.057
	PB 1st follow-up	4.3 (4.2–5.7)	3.4 (2.0–5.4)	0.29
	PB 2nd follow-up	5.2 (4.0–7.8)	3.7 (3.1–4.9)	0.083

% of ll denotes percent of lower limit for normal PB lymphocytes value.

*p<0.05, **p<0.01.

lympho, lymphocytes.

### BALF cells

Median BALF recovery was 64% (IQR 57–67) and 52% (IQR 44–65) in chronic progressive and chronic stable patients, respectively (p=0.26). BALF from chronic progressive patients disclosed higher median concentrations and percentage of lymphocytes, higher median percentage Ki-67+ and FoxP3+CD4+ T cells than patients with chronic stable disease but none of these observations were statistically significant (see table 2).

### Correlations

**Figure 1:**
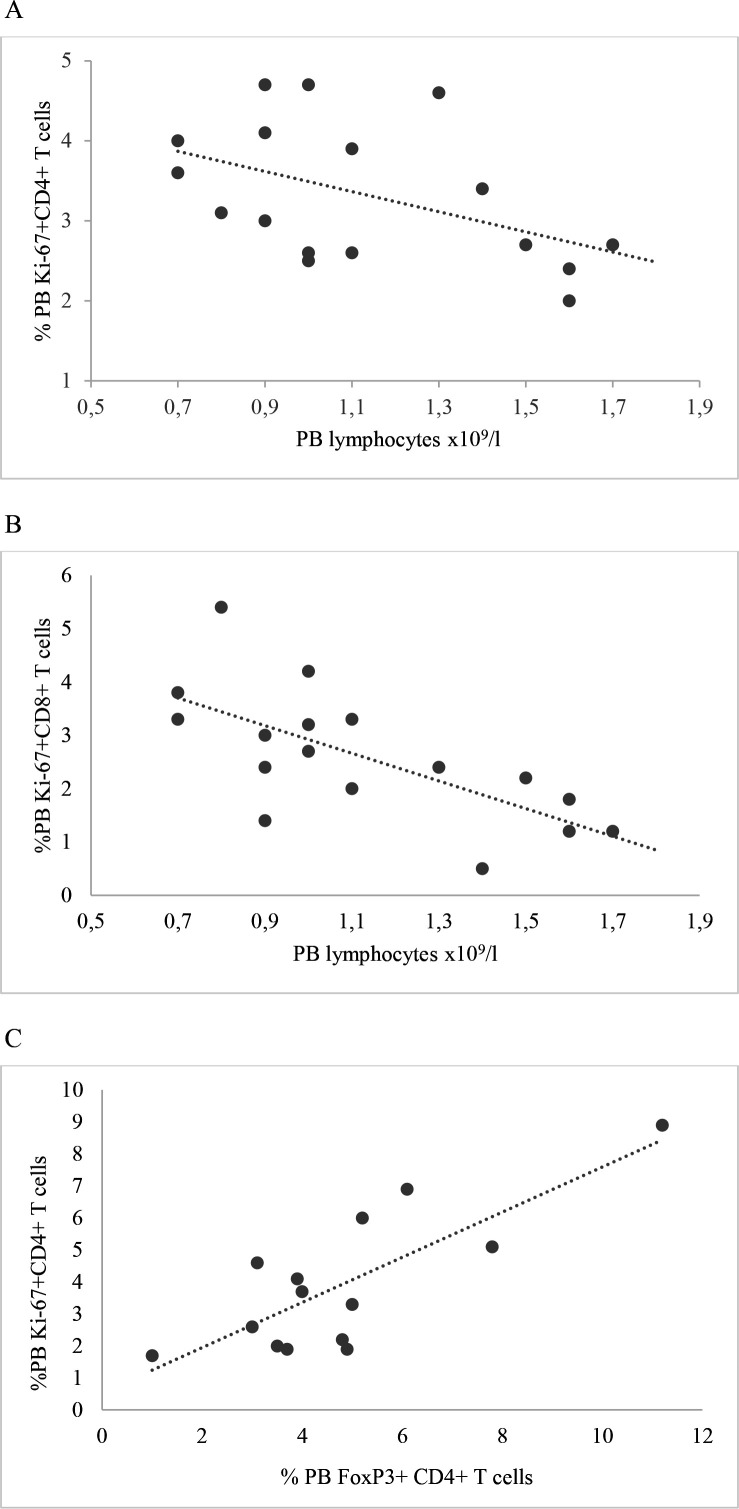
(A) Correlation between PB lymphocytes and percentage of PB CD4+T cells expressing Ki-67 at baseline, p=0.046, r=−0.49. (B) Correlation between PB lymphocytes and percentage of PB CD8+T cells expressing Ki-67 at baseline, p=0.002, r=−0.71. (C) Correlation between percentage of Ki-67+CD4+ T cells and FoxP3+CD4+ T cells at second follow-up, p=0.008, r=−0.68. PB, peripheral blood.

PB lymphocytes, neither concentration, nor percentage of lower limit, correlated with concentration or percentage BALF lymphocytes. Also, no association was found between BALF and PB lymphocytes on one hand and numbers of EPM on the other. Both the percentage of PB Ki-67+CD4+ and Ki-67+CD8+ T cells correlated negatively with concentration of PB lymphocytes at baseline (shown in figure 1A,B), while no correlations were observed at first and second follow-ups.

Percentage of PB FoxP3+CD4+ T cells correlated with percentage of PB Ki-67+CD4+ T cells at first and second follow-ups (p=0.04, r=0.5; p=0.008, r=0.8, respectively), a similar trend was also observed at baseline (p=0.1, r=0.4), data from second follow-up shown in figure 1C.

The correlations in PB described above were not observed in BALF.

## Discussion

In this study, we investigated lymphocyte numbers, percentage of proliferating CD4+ and CD8+ T cells identified by Ki-67 expression, and FoxP3+CD4+ T cells putatively representing T_regs_, in BALF and PB in patients with sarcoidosis at diagnosis, and in PB repeatedly during the first year after diagnosis. No significant differences were observed over time for any of the examined parameters. However, at several time points, differences were observed in PB between patients with chronic progressive (worsening pulmonary manifestations, increasing inflammatory activity and/or in need of systemic treatment), compared with patients with chronic stable sarcoidosis (remaining pulmonary infiltrates without deterioration, no signs of inflammatory activity and no need for systemic treatment), but not significantly in BALF. In PB, chronic progressive patients disclosed lower concentration of lymphocytes, an increased frequency of Ki-67 expressing CD4+ and CD8+ T cells, and a tendency towards higher percentage FoxP3+CD4+ T cells compared with chronic stable patients. Furthermore, the lower PB lymphocyte concentration, the higher expression of Ki-67 in both CD4+ and CD8+ T cells. In PB, the percentage of Ki-67+CD4+ T cells correlated positively with the percentage FoxP3+CD4+ T cells.

An association between PB lymphopenia and less favourable prognosis was reported already in the 70s.^14 15^ In our investigation, we found a negative correlation between percentage of PB Ki-67+CD4+ and Ki-67+CD8+ T cells on one hand, and lymphopenia on the other, indicating a depletion of PB lymphocytes despite an increased proliferation, which is in line with the hypothesis of PB lymphocytes migrating to the lung and/or extrapulmonary organs.^16 23^ Speaking against that theory though is the fact that we did not detect a correlation between either PB and BALF lymphocytes, or PB lymphocytes and numbers of EPM. It is well established that the lung lymphocytosis in sarcoidosis patients is due to a CD4+T cell expansion but the PB lymphopenia seems attributed to a general lymphocyte decrease involving not only CD4+T cells, but also CD8+T cells and CD19+B cells,^16^ which may explain the lack of correlation in our study. Furthermore, PB and BALF lymphocytes were not determined the same day, which can have led to bias. In addition, we did not actively screen for all possible EPM, thus they may be under-rated.

However, it is also possible that the PB T cell depletion is due to an increased consumption or death. One study reported an association with PB lymphopenia in sarcoidosis and under expression of certain small noncoding microRNAs involved in apoptotic pathways and genes related to lymphopenia.^24^

We did not investigate healthy controls in our study. However, the frequency of PB Ki-67+CD4+ and Ki-67+CD8+ T cells we found in the included patients with sarcoidosis is higher than 0.8%, which is the frequency for both Ki-67+CD4+ and CD8+ T cells reported from healthy controls.^25^ Thus, our finding of the lower PB lymphocyte concentration, the higher expression of Ki-67 in both CD4+and CD8+ T cells, may speak for an increased turn-over and activation of PB T cells in sarcoidosis.

In tuberculosis, another disease associated with PB lymphopenia, macrophages infected with *Mycobacterium tuberculosis* could induce T cell apoptosis.^26^ Furthermore, replicating T_regs_ are found at higher frequency in patients with more severe compared with patients with milder tuberculosis, and antigenic challenge is believed to induce peripheral T_regs_.^27 28^ Similarly, also in line with our results, Broos *et al* reported increased proportions of PB T_regs_ at diagnosis in sarcoidosis patients developing chronic disease compared with patients with spontaneous resolution and healthy controls.^11^ Taken together, it is tempting to speculate that the increased frequency of Ki-67+CD4+ and Ki-67+CD8+ T cells, as well as the tendency to increased proportion of FoxP3+CD4+ T cells we report here, reflects a compensatory mechanism for an increased T cell death due to a persistent stimulation from an unknown antigen, especially in patients with chronic active sarcoidosis. The positive correlation between Ki-67+CD4+ T cells and FoxP3+CD4+ T cells may reflect an increased proliferation of T_regs_ due to peripheral induction. It also needs to be stressed that the differences we observed between chronic progressive and chronic stable patients were not significant at every measurement. Whether this reflects timely events in the course of the sarcoid inflammation or rather is a consequence of the limited numbers of included patients, not enabling us to detect differences, is yet to be determined.

T_reg_ expansion is stimulated by TNF-α, and patients with severe and progressive sarcoidosis have higher PB TNF-α levels than patients with milder and stable disease, providing a basis for escalating expansion of T_reg._^29 30^ However, on binding to TNF-α, FoxP3 is dephosphorylated leading to suppression of T_reg_ function^31 32^ but their antiproliferative function seem intact, which is hypothesised to explain the paradoxical PB lymphopenia despite dysfunctional T_regs._^9^ Indeed, this is quite in keeping with that anti TNF-α therapy in patients with sarcoidosis, but not corticosteroids and methotrexate, is reported to increase PB lymphocyte counts, and decrease both PB and BALF T_regs_, suggesting TNF- α being of mechanistic importance.^9 33–35^

Subsequently, the findings from our investigation may indicate that measurement of PB lymphocytes, T_regs_, Ki-67+CD4+ and Ki-67+CD8+ T cells, can help to early in the disease course select patients that will benefit from intervention with TNF-α inhibitors.

In contrast to PB, this study did not show any significant differences between chronic progressive and chronic stable sarcoidosis patients in BALF. But there was a trend towards higher median absolute numbers and percentage of lymphocytes, higher median percentage of Ki-67+CD4+ and FoxP3+CD4+ T cells in patients with chronic progressive than patients with chronic stable disease. Thus, our results point in the same direction as previous findings associating higher BALF lymphocytes with non-resolving disease and increased proportions of BALF Ki-67+CD4+ T cells with disease activity.^2 3^

Besides the already mentioned lack of screening for all possible EPM, this study has some other major limitations. The relatively small study sample, some patients not participating at every follow-up, an imbalanced sex distribution, and no patients that resolved completely, limit us to draw general conclusions from the study results. Furthermore, we cannot be sure that we properly identified the T_reg_ population using FoxP3, as no definitive surface marker that uniquely isolates T_reg_ cells from other T cell populations exists. However, FoxP3 is essential for the function and has been widely used for identification of T_regs_.^36 37^

Major strengths include exploration of immune cells in two compartments, that is, lung and circulation, in treatment of naïve and phenotypically well-characterised patients, following them over a long time, enabling us to correlate immunological findings with clinical outcome.

To conclude, findings from this investigation indicate that the PB lymphopenia in patients with sarcoidosis is rather due to an increased consumption, and not sequestration in organs. Patients with PB lymphopenia should be carefully monitored as they are at risk of developing a chronic active disease, anticipating a need for treatment. Measurement of T_regs_, Ki-67+CD4+ and Ki-67+CD8+ T cells may help to early distinguish patients that will benefit from pharmacological intervention.

We now continue to collect patient data, also including patients under treatment with immunosuppressant and resolving patients. Hopefully, this will increase our understanding of T cell responses associated with clinical phenotypes, and enable us to more precisely select patients at risk for chronic active disease.

## Data Availability

Data are available on reasonable request. The data underlying this article will be shared on reasonable request to the corresponding author.
